# Using Inertial Sensors to Quantify Postural Sway and Gait Performance during the Tandem Walking Test

**DOI:** 10.3390/s19040751

**Published:** 2019-02-13

**Authors:** Kyoung Jae Kim, Yoav Gimmon, Jennifer Millar, Michael C. Schubert

**Affiliations:** 1 Department of Physical Therapy, University of Miami Miller School of Medicine, Coral Gables, FL 33146, USA; kjkim@miami.edu; 2 Neil Spielholz Functional Outcomes Research & Evaluation Center, University of Miami, Coral Gables, FL 33146, USA; 3 Department of Otolaryngology Head and Neck Surgery, Laboratory of Vestibular Neuroadaptation, Johns Hopkins University School of Medicine, Baltimore, MD 21287, USA; ygimmon1@jhmi.edu; 4 Department of Physical Medicine and Rehabilitation, Johns Hopkins University School of Medicine, Baltimore, MD 21287, USA; jmillar1@jhmi.edu

**Keywords:** tandem walking, balance, gait, body-worn inertial sensor, vestibular disorder

## Abstract

Vestibular dysfunction typically manifests as postural instability and gait irregularities, in part due to inaccuracies in processing spatial afference. In this study, we have instrumented the tandem walking test with multiple inertial sensors to easily and precisely investigate novel variables that can distinguish abnormal postural and gait control in patients with unilateral vestibular hypofunction. Ten healthy adults and five patients with unilateral vestibular hypofunction were assessed with the tandem walking test during eyes open and eyes closed conditions. Each subject donned five inertial sensors on the upper body (head, trunk, and pelvis) and lower body (each lateral malleolus). Our results indicate that measuring the degree of balance and gait regularity using five body-worn inertial sensors during the tandem walking test provides a novel quantification of movement that identifies abnormalities in patients with vestibular impairment.

## 1. Introduction

The tandem walking (TW) test is a commonly used measure of postural control during routine examinations in patients with vestibular disorders [1,2]. The patient is instructed to walk placing one foot directly in front of the other, heel-to-toe, while the examiner observes for missteps or imbalance. Patients may also be asked to repeat the test with their eyes closed.

Recently, it was reported that there is no standardized, guideline-based protocol to validate the TW test though the test remains in widespread use among clinicians [3]. During TW, altered function of the postural control system due to reduced vestibular function leads to truncal instability and irregular lurching steps that result in increased body sway, missteps, and if severe, falls [4]. Although clinicians observe for the width of the examinee’s base of support, shifts of the pelvis, or flexion of the knees, these kinematics are not quantified. Instead, the clinician qualifies the number of successful steps and/or missteps over a specified distance [5]. However, some authors suggest counting the number of successful steps during TW is not sufficiently sensitive for the assessment of short-term interval changes [6,7,8]. In theory, clinicians should be able to incorporate sophisticated equipment to measure a patient’s abnormal kinematics in addition to using their expert clinical observation. Unfortunately, such instruments are not readily available in a clinical setting. 

In the past decade, use of inertial sensor technology for balance and gait analysis has increased rapidly [9,10,11,12,13,14]. Spatio-temporal parameters measured by body-worn inertial sensors has been used in evaluating lower limb kinematics during gait in healthy adults [15,16,17,18]. The use of inertial sensors has also provided kinematic parameters that quantify balance or gait in patients with vestibular or neurological disorders. For example, instrumenting a patient with a torso-mounted inertial sensor can measure trunk kinematics (trunk angular velocity and trunk resultant acceleration) and provide a more functional indicator of balance skill during TW [1,2,19,20]. A single accelerometer worn on the lower back has been validated to assess gait characteristics in ataxic patients [21,22,23,24,25]. Recently, the TW test was instrumented using one or two inertial sensor(s) donned at the head and mid-thoracic level [1,2] in patients with vestibular pathology. This study concluded, however, that the instrumented TW test did not have high sensitivity to distinguish patients from controls. We believe that a more comprehensive kinematic approach via multiple inertial sensors might better quantify deficits in postural stability and gait performance in patients with vestibular pathology. 

Patients with vestibular-related symptoms such as vertigo and imbalance typically have increased body sway while walking [26]. The pelvis may represent the location of impaired control of the body’s center of mass. Monitoring lower limb movements during TW may also provide useful information in assessing gait regularity, which may be more irregular in patients with vestibular dysfunction due to inaccuracies processing spatial afference compared with those of the healthy individuals [26]. Recently, we quantified the range of upper body sway by measuring medio–lateral (ML) displacements at the head, trunk, and pelvis during TW [27]. We also developed a novel method to detect slight changes in gait repeatability via sensors donned at both ankles during TW [27].

The aim of the present study was therefore to provide the kinematic variables for assessing postural sway of the upper body (using three inertial sensors at the head, upper trunk, and pelvis) and gait symmetry and repeatability (using two inertial sensors at both ankles) during the TW test in healthy adults and patients with unilateral vestibular hypofunction (UVH). We hypothesized that measuring the degree of postural sway and gait regularity during the TW test using inertial sensor-determined variables would distinguish behavior in patients with UVH. 

## 2. Methods

### 2.1. Instrumentation and Preprocessing

**Figure 1:**
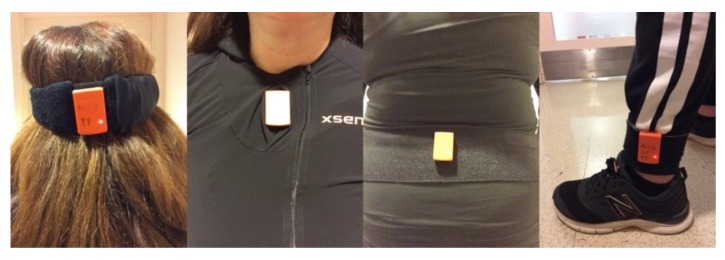
Five inertial sensors were donned on the head, upper trunk, pelvis, left and right ankles.

We used five MTw Awinda inertial sensors from Xsens Technologies (Enschede, The Netherlands, https://www.xsens.com/products/mtw-awinda/). As shown in Figure 1, the head sensor was secured to the back of the head via a headband. The trunk sensor was placed inside the pocket on the chest of an Awinda shirt (Xsens, Enschede, The Netherlands). The pelvic sensor was secured on the back at the level of the L2 vertebra above its spinous process by use of a Velcro^TM^ strap (Xsens, Enschede, The Netherlands) because the center of mass of the body lies approximately anterior to the second sacral vertebra. The ankle sensors were attached to the lateral side of the ankle just above the lateral malleolus using Velcro^TM^ straps. Each sensor comprises a tri-axial accelerometer, gyroscope, and magnetometer with respective ranges of ±160 m/s^2^, ±2000 degree/s, and ±1.9 Gauss, respectively. For this study, raw motion data (acceleration and rate of rotation along three perpendicular axes) were recorded at a sampling frequency of 100 Hz, then transmitted to the Awinda Station from Xsens Technologies and saved to a Galaxy Tab Pro S (Samsung, Korea) via USB interface. 

A fundamental problem in body-worn inertial sensor-based human motion analysis is that the sensors’ local coordinates are not aligned with any physiologically meaningful axis. For example, signals from body-worn inertial sensors are often impacted by improper mounting with respect to the body axes based on body shape or the curvature of each body segment. To compensate sensor tilt, we implemented a routine that aligned the frame of the sensor. Specifically, by using data recorded from a short standing period before the TW test, we virtually tilted the sensor’s frame of reference in alignment with the participant’s body such that gravity aligned with the x-axis corresponding to the body’s superior–inferior axis, y-axis with the ML axis, and z-axis with the anterior–posterior (AP) axis [28,29].

### 2.2. Quantification of Postural Sway

We calculated each ML displacement by double integration with high-pass filtering of the resulting acceleration from sensors donned at the head, upper trunk, and pelvis, respectively [25,30]. The amount of ML displacement was quantified by the area under the ML displacement curve [27]. In this study, an increase in the area indicated decreased upper body balance control [31]. The TW task constrains foot placement and forces increased motion variability from the upper body [1,2]. Thus, we utilized jerkiness, a measure of the rate of change in acceleration and deceleration, which reflects the amount of upper body control [32,33,34]. Sway Jerkiness was obtained using a modification of the objective function suggested by Flash and Hogan [28] and calculated as follows:

(1) $$Sway\;Jerkiness=\frac{1}{2}\int_{0}^{t}\left({\left(\frac{dx}{dt}\right)}^{2}+{\left(\frac{dy}{dt}\right)}^{2}\right)dt$$

where *x* and *y* are the acceleration data measured in the ML and AP directions, respectively. We measured the amount of jerky sway at the head, trunk, and pelvis during TW. An increase in the Sway Jerkiness indicates increased jerky sway movements (greater acceleration and velocity).

### 2.3. Quantification of Gait Regularity 

Gait regularity is commonly defined as the similarity of contralateral steps (symmetry) and the similarity of consecutive strides (repeatability) when walking on a level surface [35,36]. In this study, both gait symmetry and repeatability data were considered to evaluate abnormal limb movements during the TW test in patients with UVH. This data was captured through the two inertial sensors placed above each ankle. Specifically, we measured the sagittal plane angular velocities from each ankle (gyroscope), defined as a rotation in the sagittal plane. The data was then processed and interpreted through the dynamic time warping (DTW)-based algorithm [27] that precisely quantifies the movement difference of each gait stride signal during TW.

DTW is a commonly used technique for computing the similarity between two sequences with different length [37,38,39]. The DTW difference measure allows the identification of gait patterns without being affected by gait speed [39]. The procedures of the DTW-based method for quantifying the stride difference are as follows.

1. Segment the sagittal plane angular velocities (measured from the right and left ankle sensors) into right and left strides based on gait events (e.g., from toe-off to next toe-off) during TW. Sagittal plane angular velocity is commonly used for segmenting gait stride [40,41,42,43,44,45].
2. Calculate the right and left stride templates by averaging the segmented right and left gait stride signals, respectively. We exclude the initial and final strides from the analysis and only use the middle strides, in order to control for acceleration and deceleration influences.
3. Using the DTW method (https://www.mathworks.com/help/signal/ref/dtw.html), calculate a stride difference that is the sum of the Euclidean distances between corresponding points of gait stride signals. For example, the differences between each stride template and its consecutive stride signals were used to assess gait repeatability [27]. Gait symmetry was assessed by comparing the differences between the left and right gait stride signals.
4. Calculate symmetry and repeatability variabilities using the mean of calculated differences.

In this study, an increase in the gait regularity measure suggests decreased lower limb control. All data processing was done within MATLAB^®^ R2017b (MathWorks, Inc., MA, USA).

### 2.4. Experimental Setup

#### 2.4.1. Subjects

Fifteen subjects participated in this study (Table 1). Five old healthy adults with no vestibular symptoms, and five patients with UVH were recruited from the outpatient otolaryngology clinic at the Johns Hopkins School of Medicine. Five young healthy were also participated in this study to identify whether age affects the performance of the instrumented TW test. The identifiable vestibular pathophysiology included vestibular schwannoma, vestibular neuritis, and Meniere’s disease. The diagnosis of UVH was confirmed using abnormal video head impulse testing, abnormal videonystagmography, and an abnormal clinical examination. All study participants gave informed consent, as approved by both the Johns Hopkins University Institutional Review Board. 

#### 2.4.2. Test Procedure and Data Collection

All subjects donned five inertial sensors and began by standing in the hallway with arms folded across the chest and one foot in front of the other with heel touching toes. The subject stood still while looking straight ahead and then walked heel-to-toe 15 ft with eyes open (EO) at self-selected speed. The subject then stood still, counted to five again, and returned to the start location for their next trial. To ensure safety, an examiner guarded the subject. Each subject repeated three TW test trials with EO and three TW trials with eyes closed (EC). All sensor data recorded during testing were automatically saved to the tablet.

#### 2.4.3. Statistical Analysis

Statistical analysis was performed using SPSS (version 24, Chicago, IL, USA) software. No variables were normally distributed hence, non-parametric analysis was performed. A Related-Samples Wilcoxon Signed Rank Test was performed for each group to compare variables between conditions (EO vs EC). A Kruskal–Wallis test was performed to compare groups (young healthy controls, old healthy controls, and patients). In case of significant result of the Kruskal–Wallis test, a Dunn’s post hoc tests with Bonferroni correction were conducted for pairwise comparisons. The level of statistical significance was set at *p* ≤ 0.05. Mean and one standard deviation (1 SD) values of the dependent variables were calculated for each of the sensor data, measured during EO and EC conditions. Descriptive statistics (mean and 1 SD) were used to summarize the results. The dependent variables were: Sway Jerkiness of the Head, Trunk, and Pelvis (cm^2^/s^5^); Displacement Area of the Head, Trunk, Pelvis (cm×s); Gait Symmetry (rad/s), Gait Repeatability Left (rad/s), and Gait Repeatability Right (rad/s). 

## 3. Results

Self-selected walking speed was measured in each group. The mean (1 SD) gait velocities (ft/s) with the EO and EC conditions were 1.08 (0.21) and 0.83 (0.25) in healthy young controls, 1.11 (0.20) and 0.92 (0.21) in healthy old controls, and 0.82 (0.22) and 0.58 (0.19) in patients, respectively. The patient group were significantly slower only with the EC condition than the control groups (*p* < 0.05).

### 3.1. Postural Sway

All balance variables were significantly different between the patient group and the control groups, without any significant differences between the young and old control groups (Table 2 and Table 3). The patient group had significantly greater variables of postural sway with the EC condition than those with the EO condition (*p* < 0.05). The young and old healthy control groups had also significantly greater variables of postural sway with the EC condition than those with the EO condition (*p* < 0.05) except Displacement Area in the Pelvis (*p* > 0.1). Displacement Area in the Pelvis showed the least amount of magnitude among the Displacement Area variables during both EO and EC conditions in all groups, which means that the second sacral vertebra position did not change its position much to stay in order to maintain balance during TW. Finally, we observed that the patient group demonstrated significantly greater Sway Jerkiness variables (at the head, trunk, and pelvis) regardless of visual condition than the young and old healthy control groups.

### 3.2. Gait Regularity

All gait regularity variables were significantly different between the patient group and the young control group (Table 4 and Table 5). With the exception of gait symmetry during TW EO, there were no significant differences in gait regularity variables between the young and old control (Table 4). The significant differences between the old healthy adults and the patients were at right gait repeatability. The patient group as well as the control groups had significantly greater variables of gait regularity with the EC condition than those with the EO condition (*p* < 0.05). 

## 4. Discussion

### 4.1. Postural Sway and Gait Regularity

Balance and walking tests have been used to screen patients with UVH. It was reported that patients with UVH reduce their lateral trunk and hip translation to limit head motion over a wide base of support during normal walking [46]. A wide base of support is essential for stability because the center of mass is located well within the boundaries of the base of support. A narrowed base of support allows the center of mass to fall close to the edge of the base of support. During TW, we observed that patients with UVH could not reduce their ML sway (or side-to-side oscillation) at the upper body segments due to a narrowed base of support. Displacement Area and Sway Jerkiness variables in the patient group were therefore significantly greater from those in control group regardless of visual condition or age. Patients with UVH have also impaired control maintaining the body’s center of mass (i.e., pelvic sensor position) and display abnormally coordinated postural movement strategies. In contrast, we found no significant difference in the Displacement Area and Sway Jerkiness of the Pelvis between EO and EC in young and old healthy controls. These findings showed a strategy of the body attempting to keep the center of mass constant in healthy old adults in contrast to UVH patients with similar ages. Our results showed that Displacement Area and Sway Jerkiness variables are useful markers to evaluate abnormal postural stability due to UVH in the elderly population and why three inertial sensors on the upper body segments should be considered for assessing the motion of the upper body during TW.

The UVH patients showed significantly reduced gait repeatability of the right lower limb regardless of visual condition than the healthy old control group. The patient group also showed significantly reduced gait symmetry and repeatability compared to young healthy controls, especially when vision was absent. One of the consequences of having a vestibular disorder is that symptoms frequently cause decreased muscle strength and coordination with an increased joint stiffness [47]. Our results showed that the variable of Gait Repeatability is also a useful marker to evaluate abnormal limb movements due to UVH in the elderly population. Identifying the relationship between a specified laterality and its related gait repeatability is beyond the intent of this study. Future work with a large number of patients with UVH may provide information to clinicians with regards to this issue, however, because of the small sample we do not presume that this is currently possible.

### 4.2. Case Observation in a Patient

The TW test has been used for many years in the assessment of patients with vestibular disorders. Its efficacy as a screening tool has been controversial as well [8]. Instrumenting the TW test via a one or two body-worn inertial sensor has been introduced to make the TW test more sensitive in the clinical environment [1,2]. However, the use of one or two inertial sensor(s) on the upper body was not sensitive to screen for clinically relevant vestibular disorders. This limitation was clarified in Patient Number 3 of our study. This patient was a 40-year-old female being treated for right side vestibular neuritis. She was the youngest in the group of patients (mean 67.4 year). Her variables of Displacement Area were significantly greater compared to each of the other four patients, yet her variables of Gait Symmetry and Repeatability were the smallest. Based on the clinician’s observation, (verified from recorded video) the reason for such an apparent discrepancy appeared to be due to her effort. She worked diligently to make good steps and tried to be very accurate, which caused a greater amount of time spent in tandem stance. This also increased the motion of her upper body. In contrast, healthy controls continued to maintain balance well though they have a greater amount of time spent in tandem stance during TW than the amount of time spent in double-limb support during normal walking. This ‘gap’ between the balance and gait regularity variables was unique to this patient. If her TW performance were determined only from her increased upper body motion, she would be categorized as having severe postural instability. This was avoided in our study based on our monitoring of her gait regularity and illustrates why the TW test should be instrumented with multiple inertial sensors. 

### 4.3. Limitations

Our study population is small and the cause for UVH was varied, making generalizability ill-advised. Additionally, we recognize that controlling the level of accuracy of performance is not possible, neither is controlling the amount of effort each subject affords. However, these limitations do reflect clinical conditions and suggest that clinical observation will always be critical to qualify the objective kinematic data. Despite such limitations, our technology in this study has great potential to improve the efficiency of managing the complex balance and gait abnormalities that threaten the welfare of our healthy elderly and impaired patients [48]. We remain hopeful that data collected from different clinicians treating a variety of balance disorders coupled with a normative kinematic database would be useful to identify abnormal gait/balance performance as well as monitor change with interventions (physical therapy, medication). Although future work with a large population should be needed, our findings provide preliminary data supporting the idea that instrumenting the TW test as a measure of postural stability and gait performance can be used in patients with vestibular disorders.

## 5. Conclusions

Upper body movement and gait are two important aspects in the assessment of the tandem walking test. The present study demonstrated that the instrumented tandem walking test using five inertial sensors offers a novel assessment of postural stability and gait in patients with vestibular disorders. The present study also quantified the critical role vision has in maintaining balance and gait regularity for both healthy controls and patients with vestibular disorders. The body-worn inertial sensors and data analysis combined with the tandem walking test suggest an efficient means to distinguish abnormal behavior due to reduction or loss of the vestibular inputs.

## Figures and Tables

**Table 1:** Group characteristics.

Variable	Young Healthy Control Group	Old Healthy Control Group	Patient Group
**Number of subjects**	5	5	5
**Male/Female**	4/1	1/4	3/2
**Age (mean (1 SD))**	29.6 (5.94)	76.4 (1.67)	67.4 (16.0)
**Diagnosis**	Healthy	Healthy	Right vestibular schwannoma Right vestibular neuritis Left vestibular hypo-function Meniere’s disease (unspecified laterality) Left Meniere’s disease

SD: Standard deviation.

**Table 2:** Group comparison of postural sway variables with the eyes open condition.

Measure	Group	Mean (1 SD)	Comparison between Groups
Sway Jerkiness Head (cm^2^/s^5^)	Young control	8.13 (4.86)	*p* < 0.001
	Old control	8.57 (2.82)	
	Patient	43.96 (28.52) *#	
Sway Jerkiness Trunk (cm^2^/s^5^)	Young control	8.06 (6.02)	*p* < 0.001
	Old control	7.00 (2.69)	
	Patient	19.31 (9.03) *#	
Sway Jerkiness Pelvis (cm^2^/s^5^)	Young control	14.86 (5.55)	*p* < 0.001
	Old control	11.94 (4.21)	
	Patient	47.51 (24.96) *#	
Displacement Area Head (cm×s)	Young control	25.80 (14.25)	*p* = 0.001
	Old control	14.26 (4.64)	
	Patient	72.76 (59.09) *#	
Displacement Area Trunk (cm×s)	Young control	26.08 (13.45)	*p* < 0.001
	Old control	18.13 (5.81)	
	Patient	77.89 (60.30) *#	
Displacement Area Pelvis (cm×s)	Young control	17.13 (8.21)	*p* < 0.001
	Old control	13.77 (2.84)	
	Patient	33.62 (13.85) *#	

* Significant difference between patient group to young healthy adult group; # Significant difference between patient group to old healthy adult group.

**Table 3:** Group comparison of postural sway variables with the eyes closed condition.

Measure	Group	Mean (1 SD)	Comparison between Groups
Sway Jerkiness Head (cm^2^/s^5^)	Young control	25.38 (24.54)	*p* < 0.001
	Old control	24.38 (8.02)	
	Patient	82.69 (32.30) *#	
Sway Jerkiness Trunk (cm^2^/s^5^)	Young control	14.51 (8.92)	*p* < 0.001
	Old control	15.29 (6.20)	
	Patient	34.63 (11.92) *#	
Sway Jerkiness Pelvis (cm^2^/s^5^)	Young control	31.73 (15.91)	*p* < 0.001
	Old control	41.51 (20.09)	
	Patient	103.80 (31.83) *#	
Displacement Area Head (cm×s)	Young control	49.10 (37.76)	*p* < 0.001
	Old control	27.85 (7.16)	
	Patient	123.37 (72.92) *#	
Displacement Area Trunk (cm×s)	Young control	53.95 (37.66)	*p* < 0.001
	Old control	33.54 (9.56)	
	Patient	136.66 (85.37) *#	
Displacement Area Pelvis (cm×s)	Young control	20.91 (8.37)	*p* < 0.001
	Old control	15.87 (3.67)	
	Patient	53.56 (22.78) *#	

* Significant difference between patient group to young healthy adult group; # Significant difference between patient group to old healthy adult group.

**Table 4:** Group comparison of gait regularity variables with the eyes open condition.

Measure	Group	Mean (1 SD)	Comparison between Groups
Gait Symmetry (rad/s)	Young control	2.60 (0.78)	*p* < 0.001
	Old control	6.22 (2.54) ‡	
	Patient	11.73 (10.15) *	
Gait Repeatability Left (rad/s)	Young control	1.90 (0.80)	*p* = 0.002
	Old control	4.37 (4.00)	
	Patient	8.71 (9.42) *	
Gait Repeatability Right (rad/s)	Young control	1.83 (0.84)	*p* < 0.001
	Old control	2.94 (1.82)	
	Patient	8.15 (6.62) *#	

* Significant difference between patient group to young healthy adult group; # Significant difference between patient group to old healthy adult group; ‡ Significant difference between healthy old adult group to healthy young adult group.

**Table 5:** Group comparison of gait regularity variables with the eyes closed condition.

Measure	Group	Mean (1 SD)	Comparison between Groups
Gait Symmetry (rad/s)	Young control	9.10 (6.46)	*p* = 0.001
	Old control	13.97 (5.61)	
	Patient	21.27 (8.42) *	
Gait Repeatability Left (rad/s)	Young control	7.89 (7.54)	*p* = 0.006
	Old control	14.61 (13.58)	
	Patient	20.86 (11.56) *	
Gait Repeatability Right (rad/s)	Young control	7.36 (6.37)	*p* = 0.002
	Old control	10.97 (12.19)	
	Patient	28.63 (22.00) *#	

* Significant difference between patient group to young healthy adult group; # Significant difference between patient group to old healthy adult group.
